# Association of Dietary Vitamin K Intake With Cognition in the Elderly

**DOI:** 10.3389/fnut.2022.900887

**Published:** 2022-06-23

**Authors:** Anni Wang, Meng Zhao, Jia Luo, Tianhao Zhang, Dongfeng Zhang

**Affiliations:** ^1^Department of Epidemiology and Health Statistics, School of Public Health, Qingdao University, Qingdao, China; ^2^School of Nursing, Qingdao University Medical College, Qingdao, China

**Keywords:** cognition, vitamin K, dose–response, cross-sectional study, NHANES

## Abstract

Several previous studies discussed the association between vitamin K (VK) status and cognition. But the association between dietary VK consumption and cognitive performance in the elderly was not well understood. Therefore, we investigated the correlation between dietary VK intake and the cognition of the elderly. Our research used the data of the National Health and Nutrition Examination Survey (NHANES) from 2011 to 2014. The dietary intake of VK was assessed by two 24-h dietary recalls. The cognitive function was measured in the survey of NHANES, including the Consortium to Establish a Registry for Alzheimer’s disease Word Learning subtest (CERAD W-L), Animal Fluency Test (AFT), and Digit Symbol Substitution Test (DSST). Logistic regression and restricted cubic spline models were applied to assess the relationship between dietary VK intake and cognition. Compared with the lowest dietary VK intake group, the multivariate-adjusted odds ratio (OR) [95% confidence interval (95% CI)] of low CERAD W-L score for the highest intake group was 0.39 (0.26–0.60), the multivariate-adjusted OR (95% CI) of low AFT score was 0.59 (0.38–0.92), and the multivariate-adjusted OR (95% CI) of low DSST score was 0.44 (0.29–0.65), respectively. There was an L-shaped dose–response relationship between dietary VK intake and low CERAD W-L score. There was a linear dose–response relationship between dietary VK intake and low AFT score, and there was also a linear dose–response relationship for the low DSST score. In addition, we also found a negative association between VK from vegetables and the risk of low CERAD W-L scores. Dietary VK intake and VK intake from vegetables were inversely related to the risk of low cognitive performance of the elderly.

## Introduction

Cognitive function is associated with a variety of factors (1, 2). Many previous studies have discussed the association between nutritional factors and cognition, such as vitamin D (3, 4), zinc, copper, selenium (5), creatine (6), ω-3, and ω-6 fatty acids (7).

As a fat-soluble nutrient, vitamin K (VK) can regulate the synthesis of sphingolipids (8). As a main component of the myelin sheath and neuron membrane, sphingolipids participate in the proliferation and differentiation of neurons (9). The current studies find that VK has an important role in the nervous system (10, 11). Several previous studies have discussed the association between VK status and cognition.

For instance, a study of 31 patients with Alzheimer’s disease and 31 healthy controls found that patients had lower VK intake (12). A study of 192 geriatric patients had reported that higher dietary VK intake had a better cognitive performance (13). The results of a study of 156 elderly Irish people showed that VK status was associated with cognition (14). But in the study of 599 participants (aged 55–65 years) from the Amsterdam longitudinal aging study, VK status was not associated with cognitive performance decline (15).

At present, the association between dietary VK consumption and cognitive performance in the elderly is not well understood. The dose–response relationship and the relationship between dietary intake of VK from different sources and cognitive function has not been explored.

Therefore, the National Health and Nutrition Examination Survey (NHANES) data was used to explore the relationship between dietary VK intake and cognition of the elderly in the United States. Our research explored the dose–response relationship and sex stratification results using a larger sample size. In addition, we also explored the relationship between dietary VK intake from different sources and cognitive function.

## Materials and Methods

### Study Population

**FIGURE 1:**
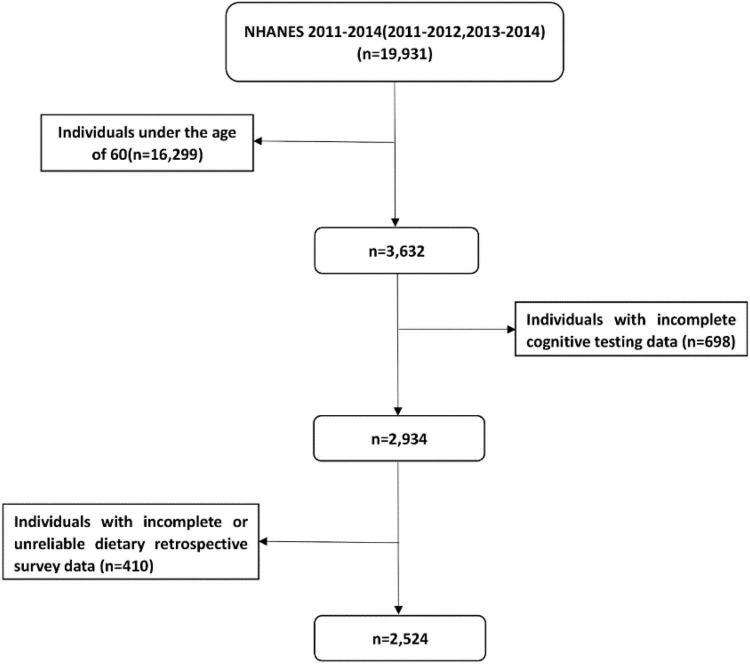
Flow chart of the screening process for the selection of eligible participants.

The data of dietary VK intake and cognitive function were obtained from two cycles (2011–2012 and 2013–2014) of the NHANES dataset. NHANES was an important plan belonging to the National Center for Health Statistics (NCHS) in the United States, and the relevant information of the survey can be obtained elsewhere (16). The 2 cycles of NHANES (2011–2012 and 2013–2014) included 19, 931 participants. We excluded participants who were under 60 years old, had incomplete cognitive test data, incomplete or unreliable dietary data. The “Dietary recall status code” in NHANES survey evaluated the quality and integrity of the dietary data. The reliable and unreliable dietary data were distinguished. Unreliable dietary data means that data on total nutrient intakes and the total foods reported in these cases were not available. These individuals had no records in the Individual Foods files (17). Finally, there were 2524 participants in our analysis, and the detailed flow chart was shown in Figure 1.

Among the 2524 participants, 1712 participants had data on VK intake from vegetables and 1450 participants had data on VK intake from grains.

### The Intake of Dietary Vitamin K

Dietary information was obtained through two 24-h dietary recall interview. Daily dietary VK intake was based on the average intake of two dietary retrospective surveys (18). A detailed description of dietary data collection can be found on the website of NHANES (19). Total VK intake data were obtained from “Total Nutrient Intakes Files,” and different sources of VK intake data were obtained from “Individual Foods Files.” Individual Foods files reported by each participant included detailed information about each food/beverage item (including the description, quantity, and nutritional ingredients). Different sources of VK intake can be identified by the Food/Individual component number (20). According to the food code of United States Department of Agriculture (USDA), we determined the intake of vegetable and grain sources VK from the dietary data (21). Referring to published articles (22), dietary VK intake was categorized into quartiles (Q1, Q2, Q3, and Q4 groups).

### Cognitive Function

There were three cognitive function tests in NHANES surveys (23). The Consortium to Establish a Registry for Alzheimer’s disease Word Learning subtest (CERAD W-L) included three consecutive learning trials and a delayed recall for new verbal information, which was used to evaluate immediate and delayed learning ability. The highest score on each test was 10. The Animal Fluency Test (AFT) was a component of executive function. To examine categorical verbal fluency, participants were asked to name as many animals as possible in 1 min, and each one scored one point. The Digit Symbol Substitution Test (DSST) assessed working memory, sustained attention, and processing speed. The score ranged from 0 to 133. In all three tests, higher scores indicated better cognitive performance. Although cognitive function tests cannot equate with clinical diagnosis, these tests proved to be good screening tools and used in research related to cognitive function (24–26).

According to the method used in the published literature (3, 5, 7, 22, 27), we further classified the scores according to three age stages. In each test, participants with scores below the minimum quartile for each age group were defined as having low poor cognitive performance. According to the cut-off points (shown in Table 1), participants were divided into low cognition performance and normal cognition performance groups.

**TABLE 1:** The cognition performance cut-off points of test score (CERAD W-L, AFT, and DSST), adjusted according to age (60–70 years, 70–80 years, and ≥80 years).

	CERAD W-L score	AFT score	DSST score
60–70 years	23	14	38
70–80 years	20	12	33
≥80 years	17	12	29

### Covariates

We included some covariates, according to the past literature on dietary factors and cognitive function (4, 27, 28). They mainly included socioeconomic status variables (such as age), health behavior variables (such as drinking status), and health factors (such as hypertension). In addition, we also adjusted the total energy intake. Detailed classification information of covariates was shown in Table 2.

**TABLE 2:** The classifications of covariates.

Covariates	Classifications
Sex	Male; female
Age (year)	60–70; 70–80; ≥80
Educational level	Below high school; high school; above high school
Marital status	Married/living with partner; widowed/divorced/separated/never married
Poverty-income ratio	≤0.99; and ≥1
Race	Mexican American; other Hispanic; non-Hispanic White; non-Hispanic Black; other race
Body mass index (BMI)	≤25 kg/m^2^; 25 to <30 kg/m^2^; ≥30 kg/m^2^
Smoked at least 100 cigarettes in life	No; yes
Had at least 12 alcohol drinks/year	No; yes
Occupational physical activity	Vigorous; moderate; other
Recreational physical activity	Vigorous; moderate; other
Hypertension^a^	No; yes
Diabetes^b^	No; yes
Stroke^c^	No; yes
Total energy intake (kcal/day)^d^	Continuous

*^a^Systolic blood pressure (SBP) ≥130 mmHg, or diastolic blood pressure (DBP) ≥80 mmHg, or ever been told by a doctor or other health professional that had hypertension, or currently taking antihypertensive drugs were hypertensive patients.*

*^b^Diabetes was defined as ever been told by a doctor or other health professional that had diabetes, or taking insulin, or fasting plasma glucose (FPG) level at least 126 mg/dL, or 2-h plasma glucose level above 200 mg/dL, or a glycated hemoglobin (HbA1c) level of 6.5% or greater.*

*^c^Stroke was defined as self-reported physician diagnosis of stroke.*

*^d^Total energy intake of each participant was the average of two 24-h dietary energy intake.*

### Statistical Analysis

According to the analysis guide of NHANES, we calculated new sample weights (29). We used the *Kolmogorov–Smirnov normality test* to test the normality of continuous variables. To describe normal variables, we used mean ± standard deviation (SD). As for non-normally distributed variables, we used the median (interquartile range) to describe it and used the *Mann–Whitney U test* to compare it. For normal distribution variables, we used the *Student’s t-test* to compare the factors between the low and normal cognition groups. The *Chi-square test* was used to compare the percentage of categorical variables.

The lowest dietary VK intake group (Q1) was taken as the reference group. Logistic regression analyses were used to explore the relationship between VK intake and cognition. We used odds ratio (OR) and 95% confidence interval (95% CI) to report results. Sex stratification analysis was also included in our study. Restricted cubic spline was used to explore the dose–response relationships between dietary VK and cognition. The three nodes were located at the 5th, 50th, and 95th percentile of dietary VK intake, respectively. We performed sensitivity analysis by excluding participants taking VK antagonists (warfarin or clopidogrel). In order to further excluded the influence of total energy intake on the results, we added a sensitivity analysis. The regression residual represented the difference between the actual VK intake for each participant and the predicted intake based on the total energy intake. The VK intake of participants was recalculated, and then regression analysis was carried out. When the *p*-value < 0.05, the results were considered statistically significant. Stata 15.0 (Stata Corp., College Station, TX, United States) was used to perform the primary statistical analysis.

## Results

### Participant’s Characteristics

For all cognitive tests, the low cognitive performance group had a lower education level, poverty-income ratio, recreational activity, total energy intake, dietary VK intake, and a higher prevalence of diabetes. For the CERAD W-L and DSST, the low cognition group had lower work physical activity level. For DSST, the low cognitive function group had a high prevalence of hypertension. For the AFT and DSST, the low cognition group had a high prevalence of stroke. The characteristics of dietary VK intake participants classified by cognitive function were shown in Table 3.

**TABLE 3:** Characteristics of the dietary VK and cognition study population, National Health and Nutrition Examination Survey (NHANES) 2011–2014 (*N* = 2524).

		CERAD W-L			AFT			DSST		
	Number of subjects (*N*)	Normal cognitive performance	Low cognitive performance	*p*-value	Normal cognitive performance	Low cognitive performance	*p*-value	Normal cognitive performance	Low cognitive performance	*p*-value
Number of subjects (%)^a^		1939 (76.82)	585 (23.18)		1952 (77.34)	572 (22.66)		1913 (75.79)	611 (24.21)	
Age (%)^a^	2524			0.3261			0.0798			0.0028
60–70 years		1030 (58.09)	338 (52.41)		1040 (57.82)	323 (53.24)		1036 (58.53)	332 (47.35)	
70–80 years		589 (27.98)	160 (32.67)		602 (28.99)	146 (27.79)		567 (28.29)	182 (32.26)	
≥70 years		320 (13.93)	87 (14.92)		310 (13.19)	95 (18.97)		310 (13.18)	97 (20.39)	
Sex^a^	2524			0.0041			0.3511			0.5684
Male		862 (44.77)	355 (55.96)		944 (46.02)	273 (50.52)		875 (46.48)	342 (48.42)	
Female		1077 (55.23)	230 (44.04)		1008 (53.98)	299 (49.48)		1038 (53.52)	269 (51.58)	
Race^a^	2524			<0.01			<0.01			<0.01
Mexican American		139 (2.67)	72 (6.80)		164 (3.11)	47 (4.90)		125 (2.43)	86 (9.98)	
Other Hispanic		159 (2.97)	85 (8.61)		168 (3.29)	76 (7.51)		124 (2.41)	120 (14.45)	
Non-Hispanic White		1046 (81.32)	223 (67.77)		1106 (82.72)	163 (58.86)		1118 (83.71)	151 (46.55)	
Non-Hispanic Black		429 (8.01)	165 (11.44)		376 (6.68)	218 (18.87)		361 (6.20)	233 (24.98)	
Other races		166 (5.04)	40 (5.37)		138 (4.20)	68 (9.86)		185 (5.25)	21 (4.05)	
Educational level (%)^a^	2475			<0.01			<0.01			<0.01
Below high school		366 (12.51)	213 (27.79)		382 (13.32)	197 (25.15)		283 (11.05)	296 (43.42)	
High school		431 (20.23)	144 (24.57)		415 (19.58)	160 (28.49)		433 (20.65)	142 (23.29)	
Above high school		1103 (67.27)	218 (47.63)		1116 (67.10)	205 (46.36)		1161 (68.30)	160 (33.29)	
Material status (%)^a^	2514			0.0444			0.0848			<0.01
Married/living with partner		1156 (67.41)	328 (61.16)		1163 (67.34)	321 (60.82)		1170 (68.43)	314 (51.93)	
Widowers/divorced /separated/never married		776 (32.59)	254 (38.84)		782 (32.66)	248 (39.18)		736 (31.57)	294 (48.07)	
Poverty-income ratio (%)^a^	2524			<0.01			<0.01			<0.01
<1		248 (6.87)	125 (14.23)		240 (6.49)	133 (17.06)		196 (5.66)	177 (25.13)	
≥1		1691 (93.13)	460 (85.77)		1712 (93.51)	439 (82.94)		1717 (94.34)	434 (74.87)	
Body mass index (%)^a^	2492			0.3840			0.9928			0.6163
<25 kg/m^2^		496 (27.24)	165 (28.02)		508 (27.42)	153 (27.15)		501 (27.05)	160 (29.64)	
25–30 kg/m^2^		660 (34.36)	206 (38.09)		677 (35.04)	189 (34.90)		671 (35.53)	195 (31.54)	
≥30 kg/m^2^		759 (38.40)	206 (33.89)		752 (37.54)	213 (37.95)		727 (37.42)	238 (38.82)	
Work activity (%)^a^	2521			<0.01			0.0857			<0.01
Vigorous		219 (14.04)	55 (9.30)		234 (13.88)	40 (9.65)		228 (13.97)	46 (8.06)	
Moderate		421 (24.46)	91 (16.19)		422 (24.07)	90 (17.31)		419 (24.09)	93 (15.66)	
Low		1297 (61.50)	438 (74.51)		1295 (62.05)	440 (73.04)		1263 (61.94)	472 (76.28)	
Recreational activity (%)^a^	2524			<0.01			<0.01			<0.01
Vigorous		209 (12.91)	34 (5.30)		210 (12.96)	33 (4.22)		229 (13.08)	14 (1.33)	
Moderate		647 (33.59)	177 (34.35)		675 (34.47)	149 (29.77)		654 (34.56)	170 (28.06)	
Low		1083 (53.49)	374 (60.36)		1067 (52.57)	390 (66.01)		1030 (52.36)	427 (70.61)	
Smoke at least 100 cigarettes in life (%)^a^	2522	971 (48.93)	304 (49.52)	0.8782	1002 (49.59)	273 (46.07)	0.3817	965 (48.59)	310 (52.01)	0.1803
Hypertension (%)^a^	2524	1553 (76.52)	472 (82.07)	0.0932	1540 (76.64)	485 (82.03)	0.0993	1508 (76.36)	517 (85.21)	0.0156
Diabetes (%)^a^	2524	585 (23.24)	217 (27.76)	0.0177	547 (22.05)	228 (34.54)	0.0013	536 (21.52)	266 (41.11)	<0.01
Stroke (%)^a^	2519	115 (5.42)	54 (8.52)	0.0524	114 (5.22)	55 (9.90)	0.0135	98 (4.97)	71 (12.72)	<0.01
Had at least 12 alcohol drinks/year (%)^a^	2506	1338 (74.12)	392 (68.12)	0.0332	1379 (75.03)	351 (62.69)	<0.01	1352 (75.04)	378 (59.66)	<0.01
Total energy intake (kcal/day)^b^	2524	1751 (831.5)	1637.5 (975.5)	0.0003	1785.75 (821.5)	1547 (866.75)	<0.01	1789 (832)	1557.5 (869.5)	<0.01
Daily dietary VK intake (mcg/day)^b^	2524	86.25 (94.35)	67.7 (76)	<0.01	86.73 (94.53)	64.88 (73.95)	<0.01	87.95 (94.2)	60.45 (73.3)	<0.01
Daily dietary VK intake from vegetable source (mcg/day)^c^	1712	42 (87.15)	35.8 (65.95)	0.0163	42 (83.65)	36.15 (67.8)	0.1604	43.95 (87.35)	31.35 (62.45)	<0.01
Daily dietary VK intake from grain source (mcg/day)^c^	1450	16.2 (21)	15.8 (20.2)	0.6944	16.7 (21.15)	13.3 (21.425)	0.0048	16.7 (21.4)	14.3 (19.95)	0.0267

*Data were the actual number of participants (weighted percentage) or medians (interquartile ranges).*

*^a^Chi-square test was used to compare the difference between normal and low cognitive performance participants.*

*^b^Mann–Whitney U test was used to compare the difference between normal and low cognitive performance participants.*

*^c^Among 2524 individuals, 1712 had completed data on VK from vegetable source, 1450 had completed VK data from grain source.*

### Association Between Dietary Vitamin K and Cognition

In the crude model, compared with the lowest dietary VK intake group, the OR (95% CI) of low CERAD W-L score for the quartile two intake group was 0.61 (0.45–0.82), for the quartile three intake group was 0.48 (0.32–0.72), and for the highest intake group was 0.27 (0.17–0.42), respectively. Compared with the lowest dietary VK intake group, the OR (95% CI) of low AFT score for the quartile three intake group was 0.53 (0.33–0.85), and for the highest intake group was 0.34 (0.23–0.52). Compared with the lowest dietary VK intake group, the OR (95% CI) of low DSST score for the quartile three intake group was 0.41 (0.29–0.57), and for the highest intake group was 0.21 (0.14–0.30).

In multi-adjusted model (model 1), compared with the lowest dietary VK intake group, the OR (95% CI) of low CERAD W-L score for the quartile two intake group was 0.67 (0.49–0.91), for the quartile three intake group was 0.63 (0.43–0.91), and for the highest intake group was 0.39 (0.26–0.60), respectively. Compared with the lowest dietary VK intake group, the OR (95% CI) of low AFT score for the highest intake group was 0.59 (0.38–0.92). Compared with the lowest dietary VK intake group, the OR (95% CI) of low DSST score for the quartile two intake group was 0.48 (0.30–0.74), and for the highest intake group was 0.44 (0.29–0.65). The joint test of the effect for the multiple categorical variables was used, and the dietary VK intake was correlated with low CERAD W-L score, with an OR value of 0.995, *p* = 0.000. For low AFT score, the OR value was 0.996, *p* = 0.001, and for low DSST score, OR value was 0.997, *p* = 0.006. The results were shown in Table 4.

**TABLE 4:** Weighted odds ratios (95% confidence intervals) for scores on CERAD W-L, AFT, DSST across dietary VK intake, NHANES 2011–2014 (*N* = 2524).

		Dietary VK intake (mcg/day)	Q1 (≤47.55)	Q2 (47.55 to ≤80.95)	Q3 (80.95 to ≤136.6)	Q4 (>136.6)
CERAD W-L	Case/participants		201/631	144/631	137/631	103/631
	Crude		1.00 (Ref.)	0.61 (0.45–0.82)*	0.48 (0.32–0.72)*	0.27 (0.17–0.42)*
	Model 1		1.00 (Ref.)	0.67 (0.49–0.91)*	0.63 (0.43–0.91)*	0.39 (0.26–0.60)*
AFT	Case/participants		197/631	157/631	120/631	98/631
	Crude		1.00 (Ref.)	1.06 (0.61–1.83)	0.53 (0.33–0.85)*	0.34 (0.23–0.52)*
	Model 1		1.00 (Ref.)	1.53 (0.81–2.90)	0.83 (0.50–1.37)	0.59 (0.38–0.92)*
DSST	Case/participants		233/631	152/631	131/631	95/631
	Crude		1.00 (Ref.)	0.36 (0.25–0.52)	0.41 (0.29–0.57)*	0.21 (0.14–0.30)*
	Model 1		1.00 (Ref.)	0.48 (0.30–0.74)*	0.72 (0.46–1.13)	0.44 (0.29–0.65)*

*Model 1 adjusted for sex, race, educational level, marital status, BMI, work physical activity, recreational physical activity, poverty-income ratio, smoking status, alcohol consumption, energy (continuous), hypertension, diabetes, and stroke.*

*Ref. refers to the reference value (Q1).*

*p < 0.05.

In sensitivity analysis, after excluding 218 participants who took VK antagonists, the association of dietary VK intake with low cognitive performance was still significant. Detailed information was shown in Supplementary Table 1. After readjusting VK intake according to total energy intake, dietary VK intake was negatively correlated with low CERAD W-L and AFT scores. Detailed information was shown in Supplementary Table 2.

In stratified analysis by sex, dietary VK intake was inversely associated with low cognitive function. In model 1, for females, compared with the lowest dietary VK intake group, the OR (95% CI) of low CERAD W-L score for the quartile two intake group was 0.38 (0.23–0.63), for the quartile three intake group was 0.52 (0.31–0.89), and for the highest intake group was 0.27 (0.15–0.48), respectively. For males, compared with the lowest dietary VK intake group, the OR (95% CI) of low AFT score for the highest intake group was 0.43 (0.19–0.96). For females, compared with the lowest dietary VK intake group, the OR (95% CI) of low DSST score for the quartile two intake group was 0.43 (0.23–0.81), and for the highest intake group was 0.42 (0.23–0.76). The results were shown in Table 5.

**TABLE 5:** Weighted odds ratios (95% confidence intervals) for scores on CERAD W-L, AFT and DSST across dietary VK intake, stratified by sex, NHANES 2011–2014 (*N* = 2524).

	CERAD W-L	Crude	Model 1	AFT	Crude	Model 1	DSST	Crude	Model 1
	Case/participants			Case/participants			Case/participants		
Male	355/1217			273/1217			342/1217		
Q1 (≤47.55)		1.00 (Ref.)	1.00 (Ref.)		1.00 (Ref.)	1.00 (Ref.)		1.00 (Ref.)	1.00 (Ref.)
Q2 (47.55 to ≤80.95)		0.87 (0.61–1.23)	1.20 (0.79–1.81)		1.43 (0.75–2.74)	2.32 (0.96–5.60)		0.35 (0.22–0.56)*	0.62 (0.31–1.23)
Q3 (80.95 to ≤136.6)		0.56 (0.36–0.87)*	0.85 (0.52–1.39)		0.74 (0.37–1.50)	1.19 (0.56–2.51)		0.50 (0.30–0.83)*	1.23 (0.63–2.37)
Q4 (>136.6)		0.39 (0.22–0.70)*	0.68 (0.35–1.30)		0.28 (0.14–0.55)*	0.43 (0.19–0.96)*		0.22 (0.13–0.36)*	0.55 (0.25–1.22)
Female	230/1307			299/1307			269/1307		
Q1 (≤47.55)		1.00 (Ref.)	1.00 (Ref.)		1.00 (Ref.)	1.00 (Ref.)		1.00 (Ref.)	1.00 (Ref.)
Q2 (47.55 to ≤80.95)		0.35 (0.22–0.56)*	0.38 (0.23–0.63)*		0.76 (0.40–1.44)	1.04 (0.50–2.15)		0.36 (0.21–0.64)*	0.43 (0.23–0.81)*
Q3 (80.95 to ≤136.6)		0.40 (0.21–0.77)*	0.52 (0.31–0.89)*		0.38 (0.19–0.75)*	0.58 (0.30–1.13)		0.33 (0.18–0.58)*	0.48 (0.22–1.06)
Q4 (>136.6)		0.17 (0.10–0.30)*	0.27 (0.15–0.48)*		0.39 (0.22–0.69)*	0.78 (0.45–1.35)		0.20 (0.12–0.35)*	0.42 (0.23–0.76)*

*Model 1 adjusted for race, educational level, marital status, BMI, work physical activity, recreational physical activity, poverty-income ratio, smoking status, alcohol consumption, energy (continuous), hypertension, diabetes and stroke.*

*Ref. refers to the reference value (Q1).*

*p < 0.05.

### Relationships Between Dietary Vitamin K Intake Derived From Different Sources (Vegetables, Grain) and the Risk of Low Cognitive Performance

The association of dietary VK intake derived from vegetables with low cognitive performance risk was shown in Table 6. In a multiple-adjusted model, dietary VK intake was correlated with low CERAD W-L score. Compared with the lowest dietary VK intake derived from vegetables group, the OR (95% CI) of low CERAD W-L score for the highest intake group was 0.60 (0.37–0.99). The joint test of the effect for the multiple categorical variables was used, with an OR value of 0.997, *p* = 0.032. We did not find the relationship between dietary VK intake from grains and the risk of low cognitive ability, and the results were shown in Table 7.

**TABLE 6:** Association between VK intake derived from vegetables and cognitive performance.

Dietary VK intake derived from vegetables (mcg/day)	Odds ratio	95% CI	*p*-value
**CERAD W-L**			
Q1 (<16.70)	1	1	
Q2 (16.70–40.85)	1.033	0.63–1.70	0.896
Q3 (40.85–99.45)	1.000	0.56–1.79	0.999
Q4 (>99.45)	0.604	0.37–0.99	0.047
**AFT**			
Q1 (<16.70)	1	1	
Q2 (16.70–40.85)	0.629	0.32–1.24	0.174
Q3 (40.85–99.45)	0.710	0.35–1.42	0.323
Q4 (>99.45)	0.629	0.37–1.06	0.081
**DSST**			
Q1 (<16.70)	1	1	
Q2 (16.70–40.85)	1.248	0.73–2.12	0.403
Q3 (40.85–99.45)	1.269	0.68–2.38	0.447
Q4 (>99.45)	0.819	0.44–1.54	0.524

**TABLE 7:** Association between VK intake derived from grain and cognitive performance.

Dietary VK intake derived from grain (mcg/day)	Odds ratio	95% CI	*p*-value
**CERAD W-L**			
Q1 (<7.90)	1	1	
Q2 (7.90–16.05)	1.581	0.94–2.66	0.082
Q3 (16.05–28.85)	1.112	0.50–2.46	0.788
Q4 (>28.85)	0.992	0.48–2.04	0.981
**AFT**			
Q1 (<7.90)	1	1	
Q2 (7.90–16.05)	0.707	0.42–1.18	0.178
Q3 (16.05–28.85)	0.752	0.49–1.14	0.176
Q4 (>28.85)	0.808	0.53–1.23	0.307
**DSST**			
Q1 (<7.90)	1	1	
Q2 (7.90–16.05)	0.939	0.56–1.58	0.809
Q3 (16.05–28.85)	0.591	0.31–1.12	0.103
Q4 (>28.85)	0.868	0.39–1.95	0.723

### Dose–Response Relationships

**FIGURE 2:**
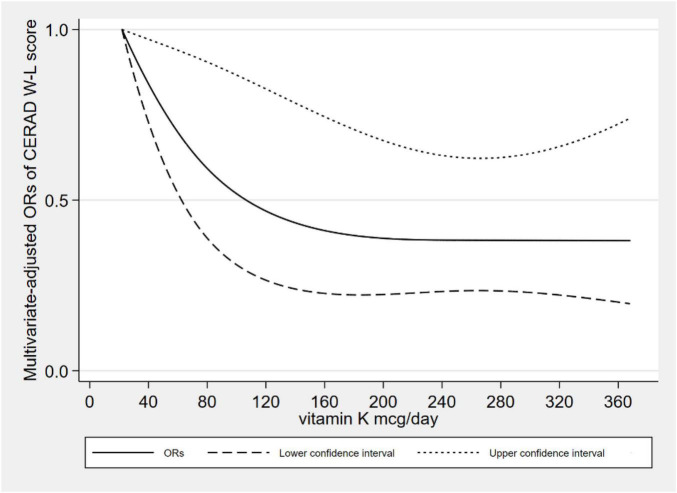
Dose–response relationship between dietary VK intake and low CERAD W-L score.

**FIGURE 3:**
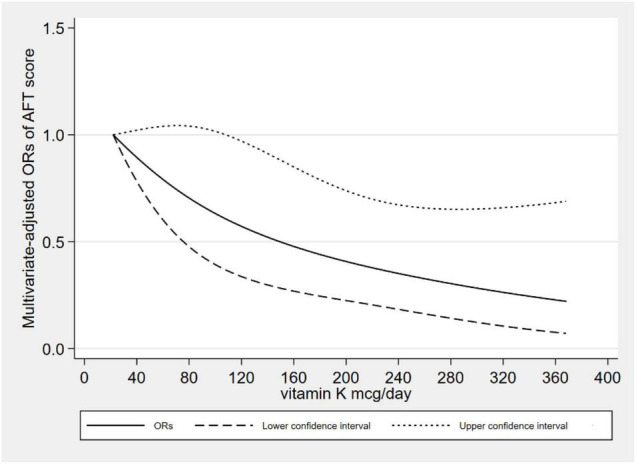
Dose–response relationship between dietary VK intake and low AFT score.

**FIGURE 4:**
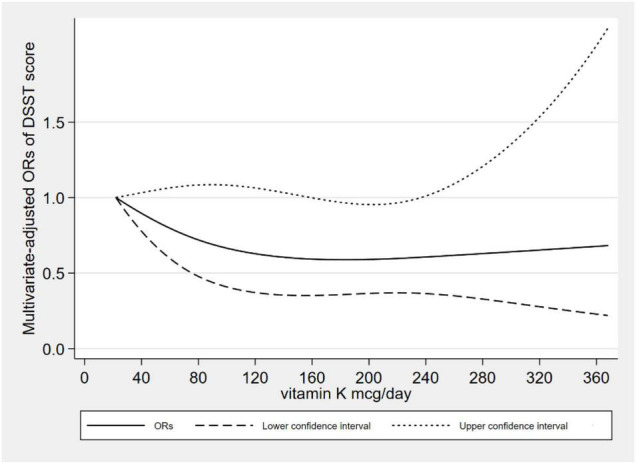
Dose–response relationship between dietary VK intake and low DSST score.

Figure 2 showed the L-shaped dose–response relationship between dietary VK intake and low CERAD W-L scores (*p*_for non–linearity_ = 0.121). It reached a plateau, when the dietary VK intake was higher than 215 mcg/day. As for dietary VK intake and low AFT scores, we found a linear dose–response relationship (*p*_for non–linearity_ = 0.664). The relationship was significant when the dietary VK intake was above 110 mcg/day (Figure 3). For the low DSST score, we found a linear dose–response relationship (*p*_for non–linearity_ = 0.325), and when dietary VK intake was lower than 163 mcg/day or higher than 234 mcg/day, the relationship was no longer significant (Figure 4).

## Discussion

There was a negative correlation between the low cognitive risk and dietary VK intake. After adjusting for many confounding factors, the correlation was still significant. For the low CERAD W-L score, we found an L-shaped dose–response relationship. This dose–response relationship means that when the plateau is reached, even if the dietary intake of VK is increased, the protective effects on cognitive function will not be increased. As for low AFT and DSST scores, we found a linear dose–response relationship, which means the prevalence of low cognitive performance decreased with increasing dietary VK intake. According to the 2020–2025 American dietary guidelines (30), the adequate intake of VK was 120 mcg for elderly males and 90 mcg for females. Of the 2524 participants, only 947 (37.52%) met the criteria.

In stratified analysis by sex, it was significant in the AFT for men and was significant in the CERAD W-L and DSST among women. We also found that the dietary VK intake derived from vegetables was negatively correlated with the low CERAD W-L score. We did not find an association between VK intake from grains and the risk of low cognitive ability, possibly due to low VK content in grains or insufficient sample size. In the sensitivity analysis, after excluding the participants who took VK antagonists, the effect of VK antagonist on the results was ruled out, and the results were still significant. According to the total energy intake, we recalculated participants’ VK intake and found that dietary VK intake was still negatively correlated with low CERAD W-L and AFT scores.

Previous studies partially explored the relationship between dietary VK intake and cognition. Some studies analyzed the clinical research evidence between VK and cognition (10). In addition, a survey conducted in France, the subjects were 160 hospitalized or seen in consultation patients (the average age was 82 years old), and the results showed that VK intake was positively correlated with the Memory Complaint Questionnaire scores (31). Another survey of 192 hospitalized or treated patients (the average age was 83 years old) in France also found higher dietary VK intake was related to better Mini-Mental State Examination scores of the elderly (13). A study of 156 elderly people (the average age was 78 years old) in Ireland showed that dietary VK was a predictor of good cognition (14). A Canadian study was conducted in 31 patients with Alzheimer’s disease and 31 healthy controls (average 78 years old) found that the intake of VK in patients with Alzheimer’s disease was lower (12). Although different dimensions of cognitive function had been evaluated, our study was consistent with previous results, and both of them found that there was an association between VK and cognitive function. A study among 599 participants (55–65 years old) showed that the association between VK status and cognition was not significant (15). This was inconsistent with our results, but we used different indicators of VK status, and our study participants were older. Such controversies may be due to the various age distribution, study design, geographical region, and methods of cognitive assessment.

There were several possible mechanisms of the relationship between dietary VK and cognitive ability (10, 32). VK played an important role in neurodegenerative diseases. VK participated in the synthesis of sphingolipids, which were the main components of myelin sheaths and were involved in the proliferation and differentiation of neurons (33). Studies showed that two VK-dependent proteins, named growth arrest-specific 6 (Gas6) and S protein, might affect the cognitive process (9). In addition, VK had anti-inflammatory activity and provided antioxidant stress protection (11).

This research had several advantages. Firstly, the dose–response relationship between dietary VK intake and cognitive performance was explored. Secondly, we used a large sample of representative American elderly data, and NHANES had a high-quality data collection method. Thirdly, we adjusted many confounding factors by referring to previous articles and made sensitivity analysis. Finally, we explored the dietary intake of VK from vegetable and grain sources.

However, this research also had some limitations. Firstly, due to the cross-sectional design, we were unable to determine a causal relationship between dietary VK intake and low cognitive risk. Second, the data obtained through 24-h dietary recall might have recall bias. Thirdly, we were unable to estimate endogenous VK production, considering only dietary VK. Finally, we can’t rule out the possible influence of other unadjusted confounding factors on the observation results.

## Conclusion

In the study, we explored the relationship between dietary VK intake and cognition. Dietary VK intake and VK intake from vegetables were inversely related to the risk of low cognitive performance of the elderly. We need further prospective studies to explore their relationship.

## Data Availability Statement

The datasets presented in this study can be found in online repositories. The names of the repository/repositories and accession number(s) can be found below: https://www.cdc.gov/nchs/nhanes/index.htm.

## Ethics Statement

The studies involving human participants were reviewed and approved by the National Center for Health Statistics Research Ethics Review Board. The patients/participants provided their written informed consent to participate in this study.

## Author Contributions

AW and MZ: conceptualization and data curation. MZ and TZ: methodology. AW and JL: writing – original draft. MZ and DZ: writing – review and editing. DZ: supervision. All authors contributed to the article and approved the submitted version.

## Conflict of Interest

The authors declare that the research was conducted in the absence of any commercial or financial relationships that could be construed as a potential conflict of interest.

## Publisher’s Note

All claims expressed in this article are solely those of the authors and do not necessarily represent those of their affiliated organizations, or those of the publisher, the editors and the reviewers. Any product that may be evaluated in this article, or claim that may be made by its manufacturer, is not guaranteed or endorsed by the publisher.
