# Supplementary material for: Association of Dietary Vitamin K Intake With Cognition in the Elderly
Source: Front Nutr. 2022 Jun 23;9:900887. doi: 10.3389/fnut.2022.900887 (PMC9260313; doi:10.3389/fnut.2022.900887)
Supplement: Supplementary file 1 [file Table_1.DOCX]

**Table S1.** Weighted odds ratios (95% confidence intervals) for scores on CERAD W-L, AFT, DSST across dietary VK intake, NHANES 2011-2014 (N=2306, after excluding 218 participants who took VK antagonists)

| **Dietary VK intake (mcg/day)** | **Odds Ratio** | **95%CI** | ***p* Value** |
| --- | --- | --- | --- |
| **CERAD W-L** |  |  |  |
| Q1(<47.70) | 1 | 1 |  |
| Q2(47.70 to 81.45)  Q3(81.45 to 138.65)  Q4(>138.65)  **AFT**  Q1(<47.70)  Q2(47.70 to 81.45)  Q3(81.45 to 138.65)  Q4(>138.65)  **DSST**  Q1(<47.70)  Q2(47.70 to 81.45)  Q3(81.45 to 138.65)  Q4(>138.65) | 0.543  0.566  0.416  1  1.453  0.865  0.614  1  0.444  0.717  0.493 | 0.38–0.78  0.39–0.82  0.27–0.65  1  0.74–2.86  0.52–1.45  0.38–1.00  1  0.26–0.76  0.49–1.05  0.33–0.74 | 0.002  0.003  0.000  0.269  0.569  0.048  0.004  0.083  0.001 |
