# Supplementary material for: Association of Dietary Vitamin K Intake With Cognition in the Elderly
Source: Front Nutr. 2022 Jun 23;9:900887. doi: 10.3389/fnut.2022.900887 (PMC9260313; doi:10.3389/fnut.2022.900887)
Supplement: Supplementary file 2 [file Table_2.DOCX]

**Table S2.** Weighted odds ratios (95% confidence intervals) for scores on CERAD W-L, AFT, DSST across dietary VK intake, NHANES 2011-2014 (N=2524, according to the total energy intake of participants, the intake of vitamin K was readjusted.)

| **Dietary VK intake (mcg/day)** | **Odds Ratio** | **95%CI** | ***p* Value** |
| --- | --- | --- | --- |
| **CERAD W-L** |  |  |  |
| Q1(<54.29) | 1 | 1 |  |
| Q2(54.29 to 87.16)  Q3(87.16 to 136.29)  Q4(>136.29)  **AFT**  Q1(<54.29)  Q2(54.29 to 87.16)  Q3(87.16 to 136.29)  Q4(>136.29)  **DSST**  Q1(<54.29)  Q2(54.29 to 87.16)  Q3(87.16 to 136.29)  Q4(>136.29) | 0.928  0.853  0.564  1  1.039  0.872  0.634  1  1.201  1.285  0.955 | 0.50–1.71  0.54–1.36  0.35–0.90  1  0.61–1.77  0.58–1.31  0.45–0.90  1  0.72–2.00  0.80–2.06  0.59–1.56 | 0.805  0.491  0.017  0.885  0.494  0.012  0.469  0.288  0.849 |
